# SOD1 Overexpression Alleviates H_2_O_2_-Induced Oxidative Stress and Reshapes Transcriptomic Responses in Chicken DF-1 Cells

**DOI:** 10.3390/antiox15091074

**Published:** 2026-08-27

**Authors:** Yidan Wang, Penghao Wei, Tinghao Gao, Zhiwei Li, Caiqin Deng, Hanjing Chen, Jianliang Zhang, Fengcai Zou, Haiyang Song

**Affiliations:** 1 Faculty of Animal Science and Technology, Yunnan Agricultural University, Kunming 650201, China; wangyidan318@163.com (Y.W.); weipenghao0414@163.com (P.W.); 2 The Yunnan Key Laboratory of Veterinary Etiological Biology, College of Veterinary Medicine, Yunnan Agricultural University, Kunming 650201, China; 19995937375@163.com (T.G.); zgtclizhiwei@163.com (Z.L.); dengcaiqin1998@163.com (C.D.); 13341572426@163.com (J.Z.); 3 College of Animal Science, Wenzhou Vocational College of Science and Technology, Wenzhou 325000, China; 15258680117@163.com

**Keywords:** SOD1, overexpression, oxidative stress, RNA-seq, DF-1 cells

## Abstract

Oxidative stress is a major consequence of environmental, metabolic, and disease-related challenges in poultry and can compromise cellular integrity and function. Although antioxidant defense mechanisms are broadly conserved across vertebrates, oxidative-stress responses in avian cells remain less well characterized than those in mammalian systems. Superoxide dismutase 1 (SOD1) is a key antioxidant enzyme, but how elevated SOD1 expression influences both oxidative injury and stress-responsive transcription in chicken cells remains incompletely understood. In this study, we established a stable SOD1-overexpressing chicken DF-1 fibroblast cell line and induced acute oxidative stress using hydrogen peroxide (H_2_O_2_). Cellular responses were evaluated using CCK-8 viability assays and Hoechst 33342/propidium iodide staining, followed by RNA sequencing and transcriptomic analyses. SOD1 overexpression markedly improved cell viability under H_2_O_2_ challenge and substantially reduced necrotic cell death, whereas its effect on apoptosis-associated changes was comparatively limited. Transcriptomic profiling showed that H_2_O_2_ induced extensive transcriptional reprogramming, while SOD1 overexpression reshaped this response by attenuating a subset of stress-inducible gene programs. Trend clustering further identified an H_2_O_2_-responsive module associated mainly with basic amino acid transport and stress-related regulatory processes that was less strongly induced in SOD1-overexpressing cells. Collectively, these findings demonstrate that elevated SOD1 expression enhances resistance to acute oxidative injury and modifies stress-responsive transcription in chicken fibroblasts. This study extends our current understanding of SOD1-mediated antioxidant cytoprotection in an avian cellular model and provides a molecular basis for further investigation of oxidative-stress regulation in poultry.

## 1. Introduction

Oxidative stress arises when the balance between oxidant production and antioxidant defense is disrupted, resulting in excessive accumulation of reactive oxygen species (ROS) [1]. Elevated ROS levels can damage major cellular components, including nucleic acids, proteins, and lipids, thereby disturbing cellular homeostasis and impairing cellular function [2,3]. In poultry production, oxidative stress is particularly relevant because multiple environmental, nutritional, and disease-associated challenges, including heat stress, high stocking density, nutritional imbalance, toxin exposure, and infection-associated inflammation, can increase ROS production and overwhelm endogenous antioxidant defenses. Persistent oxidative stress can consequently impair growth performance, immune competence, reproductive function, and overall health, making maintenance of redox homeostasis an important determinant of poultry resilience under production conditions [4,5,6].

Although the fundamental mechanisms of ROS generation and antioxidant defense are broadly conserved across vertebrates, avian species display several physiological and metabolic features that distinguish their redox biology from that of mammals. Birds generally exhibit higher mass-specific metabolic rates and blood glucose concentrations than similarly sized mammals, yet comparative studies have reported differences in cellular oxygen consumption, antioxidant capacity, and oxidative damage between the two groups [7,8,9]. These observations suggest that the relationship between metabolic activity, ROS production, and antioxidant defense in birds cannot necessarily be inferred directly from mammalian systems. In addition, modern poultry breeds are subject to substantial metabolic and environmental challenges associated with rapid growth and intensive production, further emphasizing the importance of understanding oxidative-stress regulation in avian cells.

Superoxide dismutase 1 (SOD1) is a major intracellular antioxidant enzyme that catalyzes the dismutation of superoxide radicals and plays a central role in maintaining cellular redox homeostasis [10]. Beyond its canonical antioxidant activity, increasing evidence indicates that SOD1 can also participate in broader stress-responsive processes, including redox-sensitive metabolic regulation, modulation of inflammatory signaling, and nuclear transcriptional control under oxidative stress [11]. SOD1 overexpression has been reported to reduce reactive species accumulation and attenuate downstream inflammatory responses [12], whereas SOD1 deficiency exacerbates oxidative damage and promotes pro-inflammatory immune responses [13], further supporting its importance in cellular stress regulation [14]. However, most mechanistic studies of SOD1-mediated cytoprotection and stress-responsive regulation have been conducted in mammalian systems. In comparison, how increased SOD1 abundance influences both oxidative injury and the associated transcriptional response in avian cells remains less well characterized.

We therefore hypothesized that elevated SOD1 expression would enhance the resistance of chicken cells to oxidative injury while simultaneously modifying the transcriptional programs activated during oxidative stress. To test this hypothesis, we established a stable SOD1-overexpressing chicken DF-1 fibroblast cell model and induced acute oxidative stress using hydrogen peroxide (H_2_O_2_). Cellular protection was evaluated through cell viability and cell-death analyses, while RNA sequencing was used to characterize global transcriptional responses associated with SOD1 overexpression under basal and oxidative-stress conditions. By integrating phenotypic and transcriptomic analyses, this study aimed to define how increased SOD1 expression influences oxidative-stress adaptation in an avian cellular model. These findings provide a basis for understanding SOD1-mediated antioxidant protection in chicken cells and may support future studies of redox regulation and antioxidant interventions in poultry.

## 2. Materials and Methods

### 2.1. Cell Culture

The DF-1 chicken embryo fibroblast cell line (Cat. No. iCell-c010) and HEK293T cells (Cat. No. iCell-h237) were purchased from iCell Bioscience Inc. (Shanghai, China). DF-1 chicken embryo fibroblasts and HEK293T cells were maintained in Dulbecco’s modified Eagle’s medium (DMEM; Sangon Biotech, Shanghai, China) supplemented with 10% fetal bovine serum and 1% penicillin-streptomycin at 37 °C in a humidified incubator with 5% CO_2_. Cells were passaged at 70–90% confluence and used in the logarithmic growth phase for all experiments.

### 2.2. Establishment of a Stable SOD1-Overexpressing DF-1 Cell Line

The SOD1 coding sequence used for the overexpression construct was based on the *Gallus gallus* Cu/Zn superoxide dismutase mRNA sequence deposited in GenBank under accession number U28407.1. The SOD1 overexpression construct (pLVX-SOD1-HIS-IRES-PURO) and the corresponding empty vector (NC) were packaged into lentiviral particles in HEK293T cells using psPAX2 and pCMV-VSV-G. At 70–80% confluence, 293T cells were transfected with plasmids at a ratio of pLVX-puro:pCMV-VSV-G:psPAX2 = 3:2:1 using PEI (plasmid:PEI = 1:4). Viral supernatants were collected at 24 h and 48 h post-transfection and concentrated by PEG precipitation followed by ultrafiltration. DF-1 cells were infected at 70–80% confluence with concentrated lentivirus in the presence of polybrene (8 μg/mL). After 24 h, the medium was replaced, and puromycin (1.5 μg/mL) was added at 72 h post-infection for selection. Cells were continuously selected for 14 days, and surviving cells were subjected to limiting dilution (approximately one cell per well in 96-well plates) to obtain single-cell-derived clones, which were expanded for downstream experiments.

### 2.3. PCR Verification

Genomic DNA was extracted from cells using a Blood/Cell/Tissue Genomic DNA Extraction Kit (Tiangen Biotech Co., Ltd., Beijing, China) according to the manufacturer’s instructions. DNA concentration and purity were assessed using a NanoDrop One microvolume UV-Vis spectrophotometer (Thermo Fisher Scientific, Waltham, MA, USA). DNA samples of suitable quality were used as templates for PCR amplification with primers specific for the exogenous SOD1 expression cassette. The PCR products were separated by agarose gel electrophoresis and visualized under ultraviolet illumination to verify the presence of the SOD1 expression cassette.

### 2.4. RT-qPCR Analysis

Total RNA was extracted using TRIzol reagent (Thermo Fisher Scientific, Waltham, MA, USA) according to the manufacturer’s instructions. RNA concentration and purity were assessed by spectrophotometry. First-strand cDNA was synthesized using a commercial reverse transcription kit following the manufacturer’s protocol. Quantitative PCR was performed using PerfectStart Green qPCR SuperMix (TransGen Biotech, Beijing, China) on a real-time PCR system. GAPDH was used as the internal reference gene, and relative gene expression was calculated using the 2^−ΔΔCt^ method. Each assay was performed with at least three biological replicates. The primer sequences used for RT-qPCR are listed in Supplementary Table S1.

### 2.5. Western Blotting

Total protein was extracted using RIPA lysis buffer (Beyotime Biotechnology, Shanghai, China) supplemented with protease inhibitors. Protein concentration was determined using a BCA protein assay kit (Beyotime Biotechnology, Shanghai, China). Equal amounts of protein were separated by SDS-PAGE (Beyotime Biotechnology, Shanghai, China) and transferred onto PVDF membranes. After blocking with 5% nonfat milk (Solarbio Science & Technology, Beijing, China), the membranes were incubated with primary antibodies against SOD1 and GAPDH, with GAPDH used as the loading control. The membranes were then incubated with HRP-conjugated secondary antibodies. Protein signals were detected using an enhanced chemiluminescence system and quantified by densitometric analysis.

### 2.6. Hydrogen Peroxide-Induced Oxidative Stress Model

Hydrogen peroxide (H2O2; 3% stock solution, Guangdong Hengjian Pharmaceutical, Foshan, China) was diluted in complete medium immediately before use. For dose-time viability profiling, DF-1 cells (NC and OE-SOD1) were exposed to H_2_O_2_ at 0, 600, 700, 800, 900, or 1000 μM for 1, 2, or 3 h. Because H_2_O_2_ sensitivity varies among cell types, we first performed dose-time viability screening in DF-1 cells. Previous studies have used H_2_O_2_ to induce oxidative injury in DF-1 cells and other chicken-derived cell models [15,16]. Based on our screening results, 1000 μM H_2_O_2_ for 3 h was selected to induce robust acute oxidative stress.

### 2.7. Cell Viability Assay

Cell viability was assessed using an enhanced Cell Counting Kit-8 (CCK-8) (Beyotime Biotechnology, Shanghai, China). Cells were seeded in 96-well plates and allowed to adhere overnight. After H_2_O_2_ treatment, the medium was replaced with fresh medium containing CCK-8 reagent (10 μL per 100 μL medium) and incubated for 3 h at 37 °C. Absorbance was measured at 450 nm using a microplate reader. Cell viability was calculated as: Cell viability (%) = [(A_s_ − A_β_)/(A_c_ − A_β_)] × 100, where A_s_ is the absorbance of treated wells, A_c_ is the absorbance of untreated control wells, and A_β_ is the absorbance of blank wells. Each condition was tested with three biological replicates. Statistical analysis was performed using one-way ANOVA in SPSS software (v.29.0; https://www.ibm.com/products/spss-statistics; accessed on 21 June 2026), and a *p* < 0.05 was considered statistically significant.

### 2.8. Assessment of Apoptosis and Necrosis by Hoechst/PI Staining

Apoptosis and necrosis were assessed using an Apoptosis and Necrosis Assay Kit (Beyotime Biotechnology, Shanghai, China). After treatments, cells were washed with PBS and stained with Hoechst 33342 and propidium iodide (PI) according to the manufacturer’s instructions. Images were acquired using an inverted fluorescence microscope (ECLIPSE Ts2-FL; Nikon Corporation, Tokyo, Japan). For signal interpretation, normal cells were defined as exhibiting weak red fluorescence (PI) together with weak blue fluorescence (Hoechst 33342); apoptotic cells were defined as exhibiting weak red fluorescence together with strong blue fluorescence (bright/condensed nuclei); and necrotic cells were defined as exhibiting strong red fluorescence together with strong blue fluorescence. Quantification was performed by counting cells in randomly selected fields for each biological replicate. Fluorescence intensity was quantified using ImageJ (v1.53; https://imagej.net/ij/index.html; accessed on 21 June 2026), and apoptosis/necrosis indices were calculated after background subtraction.

### 2.9. RNA Extraction, Library Preparation, and Sequencing

For RNA-seq, four experimental groups were analyzed: NC, OE-SOD1, NC + H_2_O_2_ (1000 μM, 3 h), and OE-SOD1 + H_2_O_2_ (1000 μM, 3 h), with three biological replicates per group. RNA quality was evaluated before library construction. RNA integrity and potential DNA contamination were assessed by agarose gel electrophoresis, RNA purity was examined using a NanoDrop spectrophotometer based on the OD260/280 and OD260/230 ratios, and RNA integrity was further evaluated using an Agilent 2100 Bioanalyzer (Agilent Technologies, Santa Clara, CA, USA). mRNA was enriched using mRNA Capture Beads and fragmented at high temperature after bead purification. The fragmented mRNA was used as the template for first-strand cDNA synthesis, followed by second-strand cDNA synthesis, during which end repair and A-tailing were performed. Sequencing adapters were subsequently ligated, and fragments of the desired size were purified using Hieff NGS^®^ DNA Selection Beads (Yeasen Biotechnology, Shanghai, China). The libraries were amplified by PCR and subjected to sequencing on the Illumina NovaSeq X Plus platform.

### 2.10. RNA-Seq Quality Control and Read Mapping

Raw sequencing reads were subjected to quality control using fastp [17] (v0.18.0; https://github.com/OpenGene/fastp; accessed on 21 June 2026). Reads containing sequencing adapters, reads with more than 10% ambiguous bases (N), reads consisting entirely of adenine bases, and low-quality reads in which bases with quality scores ≤20 accounted for more than 50% of the read length were removed. The remaining high-quality reads were retained as clean reads for downstream analyses.

To remove residual ribosomal RNA (rRNA) reads, clean reads were aligned against the corresponding rRNA database using Bowtie2 [18] (v2.2.8; Bowtie2 website; accessed on 21 June 2026) without allowing mismatches, and reads mapped to rRNA were discarded. The remaining paired-end reads were mapped to the *Gallus gallus* reference genome using HISAT2 [19] (v2.1.0; https://daehwankimlab.github.io/hisat2/; accessed on 21 June 2026) with default parameters.

### 2.11. Quantification of Gene Abundance

Based on the HISAT2 alignment results, transcripts were reconstructed using StringTie [20,21] (v1.3.1; https://ccb.jhu.edu/software/stringtie/; accessed on 21 June 2026). Gene expression abundance in each sample was subsequently quantified using Salmon [22] (v1.10.3; https://combine-lab.github.io/salmon/; accessed on 21 June 2026). Gene expression levels were represented by raw read counts and fragments per kilobase of transcript per million mapped reads (FPKM). Raw read counts represented the number of sequencing fragments assigned to each gene and were used as input for differential expression analysis. FPKM values were normalized for both sequencing depth and gene length and were used to describe relative gene-expression abundance and for subsequent visualization analyses.

### 2.12. Principal Component Analysis

Principal component analysis (PCA) was performed in R (R Project for Statistical Computing; http://www.r-project.org/; accessed on 21 June 2026) based on gene-expression abundance data. PCA was used to reduce the dimensionality of the expression matrix and evaluate the overall relationships among samples. The first two principal components were used to visualize the distribution of biological replicates and experimental groups, with samples exhibiting more similar global expression profiles positioned closer together in the PCA space. Sample-to-sample correlation analysis was also performed in R. Pearson correlation coefficients were calculated between all pairs of samples to evaluate the reproducibility of biological replicates and the similarity of global gene-expression profiles among experimental conditions.

### 2.13. Differentially Expressed Genes

Differential gene expression analysis was performed using DESeq2 [23] (v1.24; https://bioconductor.org/packages/release/bioc/html/DESeq2.html; accessed on 21 June 2026) based on raw gene-level read counts. Because each experimental condition contained three biological replicates, DESeq2 was used for all pairwise comparisons. The differential expression analysis consisted of three major steps: normalization of read counts to account for differences in sequencing depth among samples, statistical hypothesis testing for differences in gene expression between experimental groups, and multiple-testing correction to control the false discovery rate (FDR). Genes with FDR < 0.05 and |log_2_ fold change| ≥ 2 were considered significantly differentially expressed genes (DEGs).

Four pairwise comparisons were analyzed: OE-SOD1 versus NC, NC + H_2_O_2_ versus NC, OE-SOD1 + H_2_O_2_ versus OE-SOD1, and OE-SOD1 + H_2_O_2_ versus NC + H_2_O_2_. Hierarchical clustering of DEGs was performed after z-score transformation of gene-expression values, and the resulting expression patterns were visualized using heatmaps.

### 2.14. GO and KEGG Functional Enrichment Analyses

GO and KEGG enrichment analyses were performed for DEGs using the Gene Ontology database [24] (https://geneontology.org/; accessed on 21 June 2026) and the KEGG database [25] (https://www.genome.jp/kegg/; accessed on 21 June 2026), respectively. Enrichment significance was evaluated using hypergeometric tests by comparing the number of DEGs assigned to each GO term or KEGG pathway with the corresponding number of annotated genes in the background gene set. For GO analysis, *p* values were corrected using the Bonferroni method, and terms with corrected *p* ≤ 0.05 were considered significantly enriched. For KEGG analysis, pathways with a Q value ≤ 0.05 after multiple-testing correction were considered significantly enriched. Dot plots, functional-category summaries, and enrichment networks were generated to visualize the major enriched biological processes and pathways.

### 2.15. Protein–Protein Interaction

Protein–protein interaction (PPI) analysis was performed using STRING [26] (v10; https://string-db.org/; accessed on 21 June 2026). Proteins encoded by the genes of interest were mapped to the interaction information available in the STRING database, and known and predicted protein associations were retrieved to construct the PPI network. The resulting interaction network was visualized using Cytoscape [27] (v3.7.1; https://cytoscape.org/; accessed on 21 June 2026). Proteins were represented as nodes and protein–protein associations as edges. Node connectivity was used to visualize highly connected proteins and major interaction modules within the analyzed gene set.

## 3. Results

### 3.1. Generation and Validation of SOD1-Overexpressing DF-1 Cells

**Figure 1:**
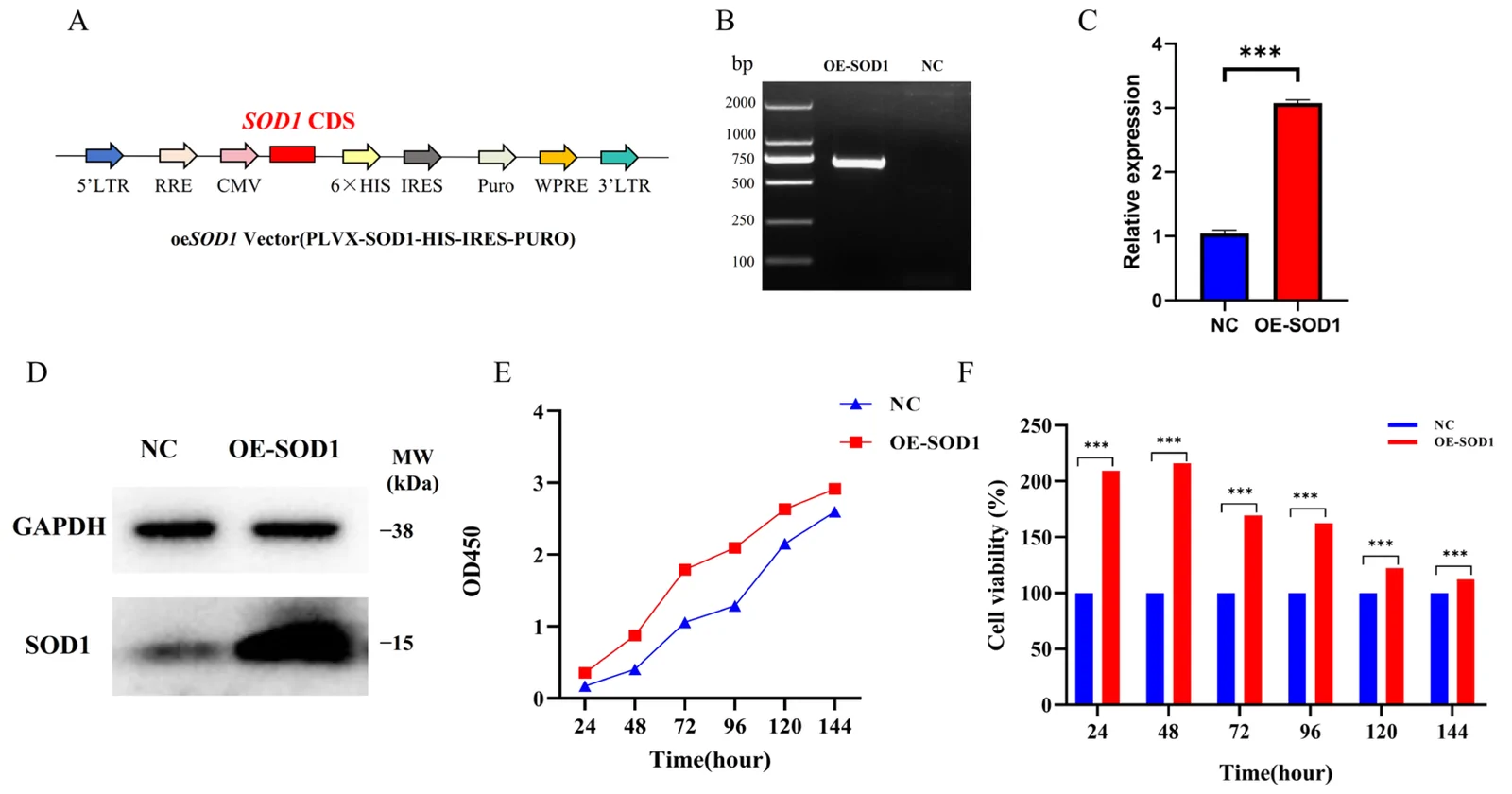
Generation and validation of SOD1-overexpressing DF-1 cells. (**A**) Schematic diagram of the lentiviral SOD1 overexpression construct (PLVX-SOD1-HIS-IRES-PURO). (**B**) Genomic PCR verification of transgene integration in OE-SOD1 cells. (**C**) RT-qPCR quantification of SOD1 mRNA expression in NC and OE-SOD1 cells. (**D**) Western blot analysis of SOD1 protein levels in NC and OE-SOD1 cells; GAPDH served as a loading control. (**E**) Growth curves measured by CCK-8 assay (OD450) over a 24–144 h time course. (**F**) Relative cell viability normalized to NC at each time point. Data are presented as mean ± SD. *** indicates *p* < 0.001.

To establish a stable DF-1 cell line with enforced SOD1 expression, the SOD1 coding sequence was cloned into a lentiviral overexpression vector under the control of the CMV promoter. The construct contained a C-terminal 6×His tag and an IRES-puromycin selection cassette (pLVX-SOD1-His-IRES-Puro; Figure 1A). Following lentiviral transduction and puromycin selection, genomic integration of the exogenous SOD1 cassette was verified by PCR. A single band of the expected size of 700 bp was detected in OE-SOD1 cells, whereas no corresponding amplification was observed in control cells (Figure 1B).

We next confirmed SOD1 overexpression at both the mRNA and protein levels. RT-qPCR analysis showed that SOD1 transcript abundance was significantly higher in OE-SOD1 cells than in negative control (NC) cells (Figure 1C; *p* < 0.001). Consistently, Western blot analysis revealed a marked increase in SOD1 protein expression in OE-SOD1 cells, with GAPDH used as the loading control (Figure 1D).

Because stable overexpression of SOD1 might also influence basal cellular growth or fitness, we further assessed cell growth under routine culture conditions. CCK-8 assays showed that OE-SOD1 cells exhibited consistently higher OD450 values than NC cells throughout the 24–144 h observation period (Figure 1E). After normalization to NC cells, relative cell viability remained significantly higher in the OE-SOD1 group at all examined time points (Figure 1F; *p* < 0.001).

### 3.2. SOD1 Overexpression Confers Resistance to H_2_O_2_-Induced Oxidative Stress in DF-1 Cells

**Figure 2:**
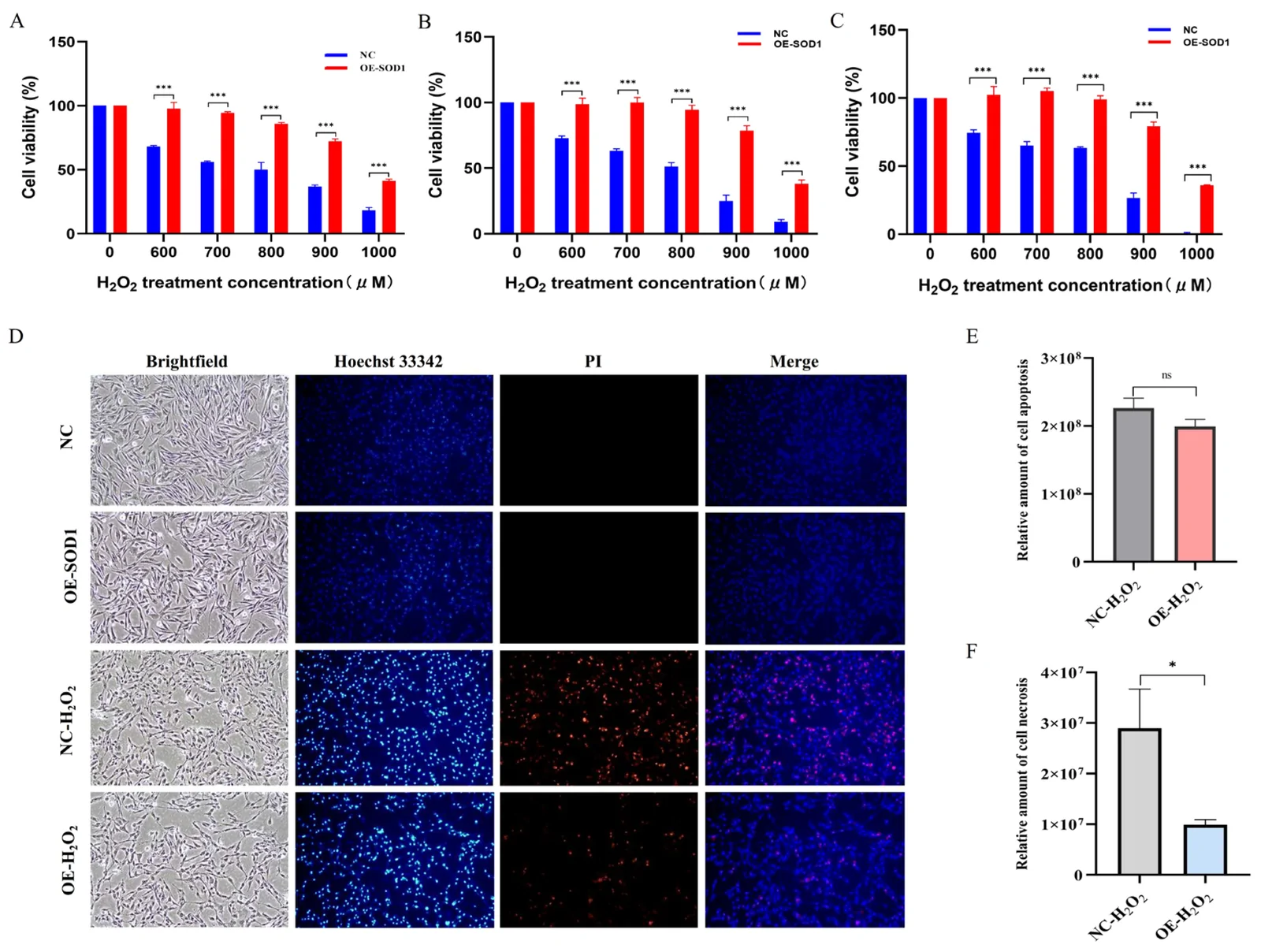
SOD1 overexpression attenuates H_2_O_2_-induced cytotoxicity and necrotic cell death in DF-1 cells. (**A**–**C**) CCK-8 viability of NC and OE-SOD1 DF-1 cells following exposure to increasing concentrations of H_2_O_2_ (0–1000 μM) for 1 h (**A**), 2 h (**B**), and 3 h (**C**). (**D**) Representative images of Hoechst 33342/PI staining in untreated cells and in cells treated with 1000 μM H_2_O_2_ for 3 h. (**E**,**F**) Quantification of apoptosis (**E**) and necrosis (**F**) based on Hoechst and PI signals, respectively. ns indicates no significant difference between groups; * indicates *p* < 0.05; *** indicates *p* < 0.001.

To establish an acute oxidative stress model and evaluate the cytoprotective effect of SOD1, NC and OE-SOD1 DF-1 cells were exposed to increasing concentrations of H_2_O_2_ (0–1000 μM) for 1–3 h, followed by CCK-8 analysis. H_2_O_2_ induced a concentration-dependent decrease in cell viability in both groups. However, OE-SOD1 cells consistently maintained higher viability than NC cells under oxidative challenge at 1 h (Figure 2A), 2 h (Figure 2B), and 3 h (Figure 2C).

We next examined whether SOD1 overexpression affected the mode of cell death under severe oxidative stress. Cells were treated with 1000 μM H_2_O_2_ for 3 h and then subjected to Hoechst 33342/propidium iodide (PI) dual staining. H_2_O_2_ exposure markedly increased nuclear condensation and/or fragmentation, indicative of apoptosis-associated morphological changes, as well as PI-positive staining, indicative of membrane integrity loss (Figure 2D). Compared with NC cells, OE-SOD1 cells showed a visibly lower proportion of PI-positive cells under the same H_2_O_2_ treatment, suggesting that SOD1 overexpression attenuated membrane-disruptive cell death. Quantitative analysis further supported these observations. The Hoechst-based apoptosis readout showed no significant difference between NC and OE-SOD1 cells under the applied oxidative stress condition (Figure 2E). In contrast, the PI-based necrosis readout was significantly reduced in OE-SOD1 cells compared with NC cells (Figure 2F).

### 3.3. Global Transcriptomic Landscape of DF-1 Cells Under Oxidative Stress

**Figure 3:**
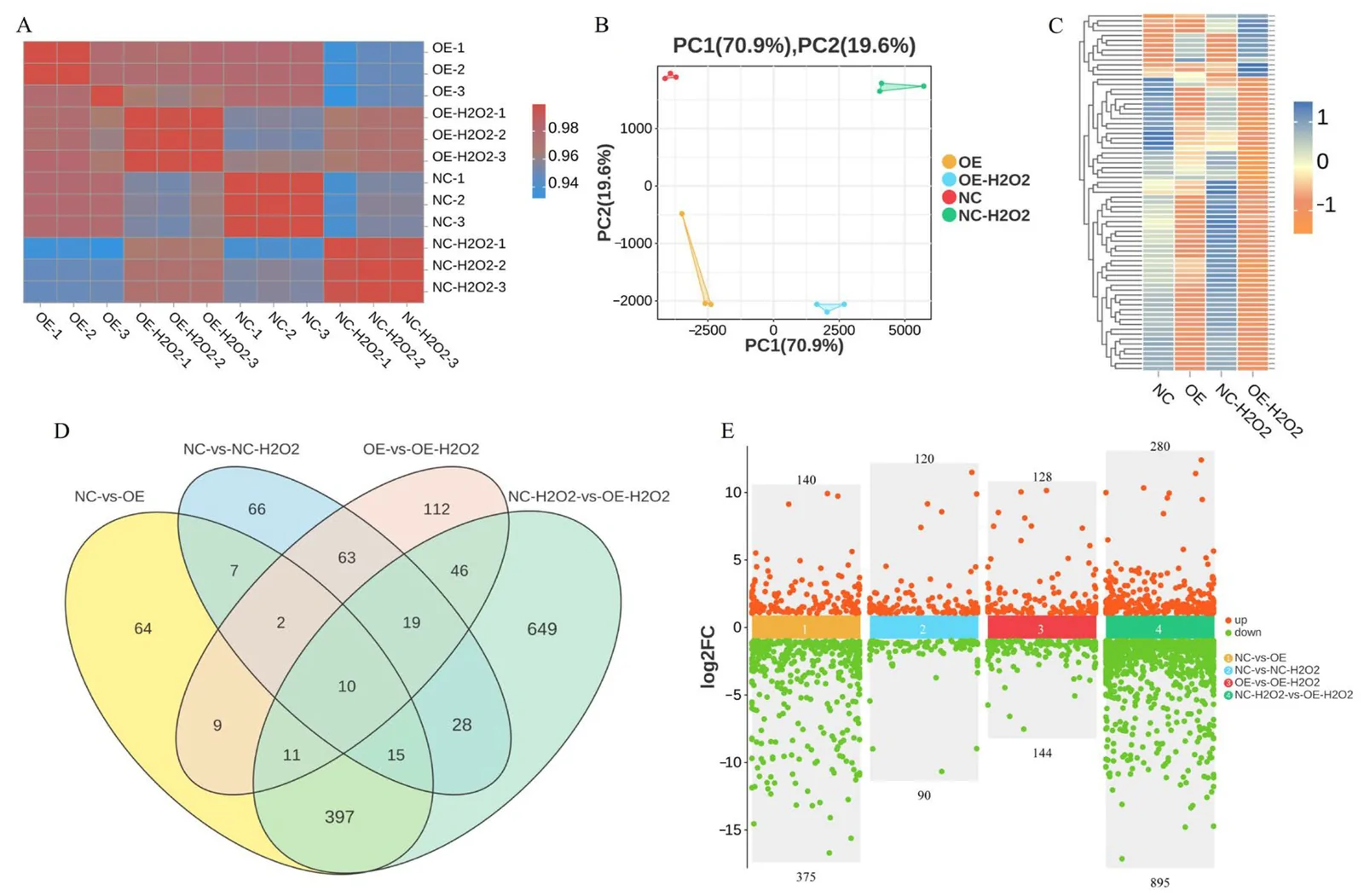
Global transcriptional landscape of DF-1 cells under oxidative stress. (**A**) Sample correlation heatmap of RNA-seq expression profiles across four conditions (NC, OE-SOD1, NC + H_2_O_2_, and OE-SOD1 + H_2_O_2_; *n* = 3 per group), with hierarchical clustering indicating high within-group reproducibility. (**B**) Principal component analysis (PCA) showing clear separation of the four experimental groups. (**C**) Unsupervised heatmap of differentially expressed genes across the four conditions (row-scaled z-scores), illustrating condition-dependent transcriptional patterns. (**D**) Venn diagram showing overlap of DEGs among the four pairwise comparisons (OE vs. NC, NC-H_2_O_2_ vs. NC, OE-H_2_O_2_ vs. OE, and OE-H_2_O_2_ vs. NC-H_2_O_2_). (**E**) Overview of differentially expressed genes in each comparison, showing the distribution of upregulated (red) and downregulated (green) genes.

To characterize the transcriptomic responses associated with SOD1 overexpression and oxidative stress, RNA-seq was performed on four experimental groups: NC, OE-SOD1, NC + H_2_O_2_, and OE-SOD1 + H_2_O_2_. Sample correlation analysis showed high similarity among biological replicates within each group and clear separation between different groups, indicating good reproducibility and distinct transcriptional states across the four conditions (Figure 3A). Consistently, principal component analysis (PCA) revealed clear clustering of the four experimental groups, with PC1 and PC2 explaining 70.9% and 19.6% of the total variance, respectively (Figure 3B).

Unsupervised clustering of differentially expressed genes (DEGs) further revealed condition-specific transcriptional patterns across the four groups (Figure 3C). Differential expression analysis identified extensive transcriptomic changes among pairwise comparisons, and Venn analysis showed both shared and comparison-specific DEG sets across the four contrasts (Figure 3D). The numbers and proportions of upregulated and downregulated genes varied across comparisons, with the largest transcriptional shifts observed between H_2_O_2_-treated groups and their corresponding untreated controls (Figure 3E). Genotype-dependent differences were also evident under both basal and oxidative stress conditions, indicating that SOD1 overexpression reshaped the transcriptional landscape of DF-1 cells before and after oxidative challenge.

### 3.4. Functional Enrichment Analysis of DEGs

**Figure 4:**
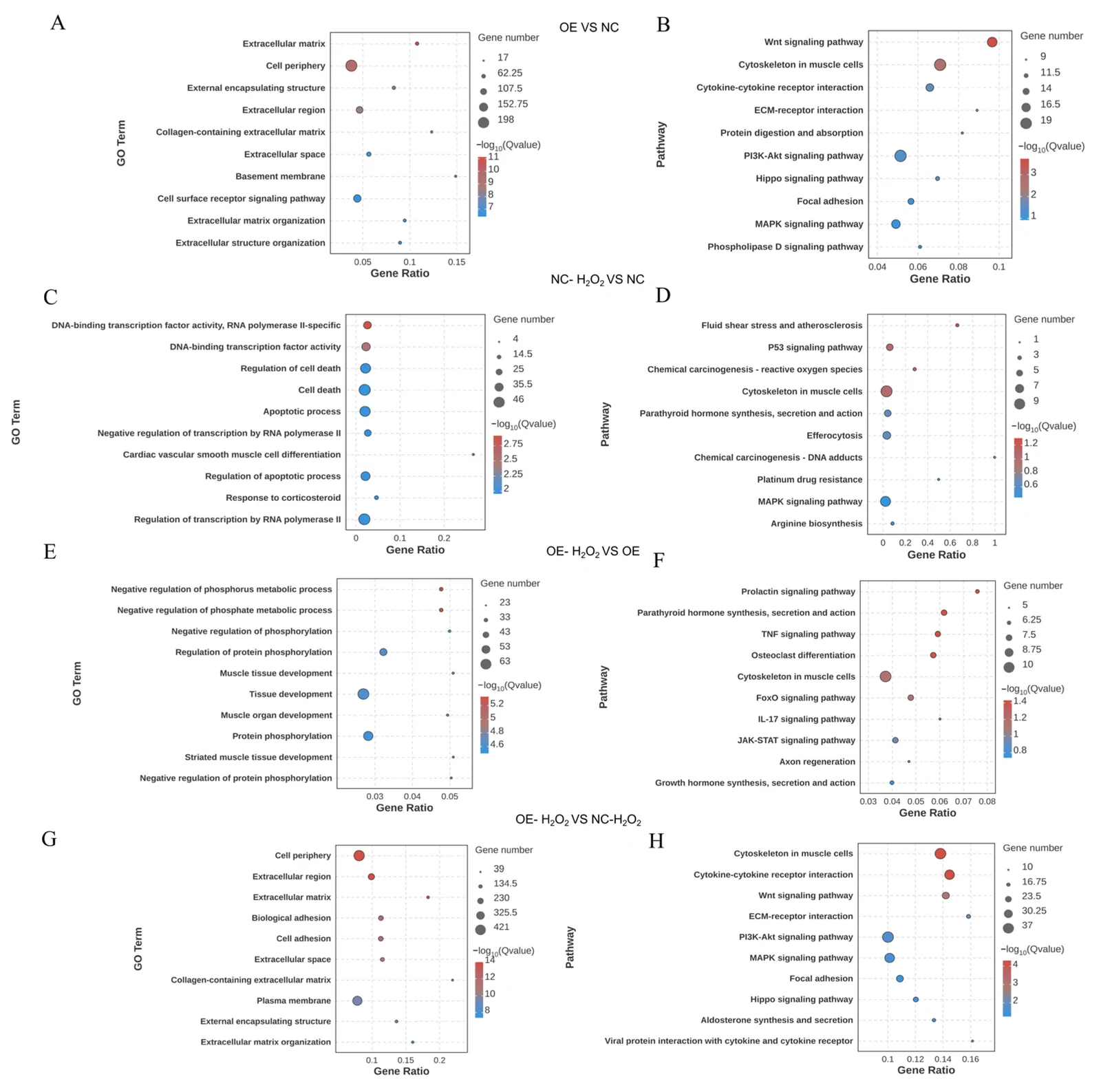
GO and KEGG enrichment analyses of DEGs across four contrasts. (**A**,**B**) Enrichment results for OE vs. NC. (**A**) Top 10 enriched GO terms. (**B**) Top 10 enriched KEGG pathways. (**C**,**D**) Enrichment results for NC-H_2_O_2_ vs. NC. (**C**) Top 10 enriched GO terms. (**D**) Top 10 enriched KEGG pathways. (**E**,**F**) Enrichment results for OE-H_2_O_2_ vs. OE. (**E**) Top 10 enriched GO terms. (**F**) Top 10 enriched KEGG pathways. (**G**,**H**) Enrichment results for OE-H_2_O_2_ vs. NC-H_2_O_2_. (**G**) Top 10 enriched GO terms. (**H**) Top 10 enriched KEGG pathways. Dot size indicates the number of DEGs mapped to each term/pathway, dot color denotes −log10(q value), and the x-axis shows gene ratio (mapped DEGs/total genes in the term).

To interpret the biological processes underlying the transcriptional differences among the four experimental conditions, GO and KEGG enrichment analyses were performed for DEGs from each pairwise comparison. The top 10 enriched terms and pathways are summarized in Figure 4. In the baseline comparison between OE-SOD1 and NC cells, GO enrichment was mainly associated with extracellular and cell–matrix-related categories, including extracellular matrix, collagen-containing extracellular matrix, extracellular region/space, and extracellular structure organization (Figure 4A). Consistently, KEGG analysis highlighted pathways related to cell–matrix interaction and signal transduction, including Wnt signaling, cytokine–cytokine receptor interaction, ECM–receptor interaction, focal adhesion, and major signaling pathways such as PI3K-Akt, Hippo, and MAPK signaling (Figure 4B).

For H_2_O_2_-induced transcriptional changes in NC cells, based on the NC + H_2_O_2_ versus NC comparison, GO enrichment was primarily related to stress-associated transcriptional regulation and cell fate processes, including DNA-binding transcription factor activity, regulation of cell death, and apoptotic processes (Figure 4C). KEGG enrichment further identified multiple stress- and injury-related pathways, including p53 signaling, chemical carcinogenesis-reactive oxygen species, chemical carcinogenesis-DNA adducts, MAPK signaling, and efferocytosis (Figure 4D).

For H_2_O_2_-induced changes in the SOD1-overexpressing background, based on the OE-SOD1 + H_2_O_2_ versus OE-SOD1 comparison, GO terms were enriched for phosphorylation-related regulatory processes, including negative regulation of phosphorylation, negative regulation of protein phosphorylation, and phosphatase-associated processes (Figure 4E). KEGG enrichment mapped these DEGs to multiple signaling pathways, including TNF signaling, FoxO signaling, JAK-STAT signaling, IL-17 signaling, and osteoclast differentiation, indicating broad signal-regulatory involvement under oxidative stress in OE-SOD1 cells (Figure 4F).

Finally, the genotype comparison under oxidative stress, OE-SOD1 + H_2_O_2_ versus NC + H_2_O_2_, again showed GO enrichment dominated by extracellular and adhesion-related categories, including cell periphery, extracellular region, extracellular matrix, cell adhesion, and collagen-containing extracellular matrix (Figure 4G). Correspondingly, KEGG analysis highlighted pathways associated with cell–matrix interaction and major signaling networks, including cytokine–cytokine receptor interaction, ECM–receptor interaction, PI3K-Akt signaling, MAPK signaling, focal adhesion, and Hippo signaling (Figure 4H).

### 3.5. STEM Clustering Identifies Distinct Expression Programs

**Figure 5:**
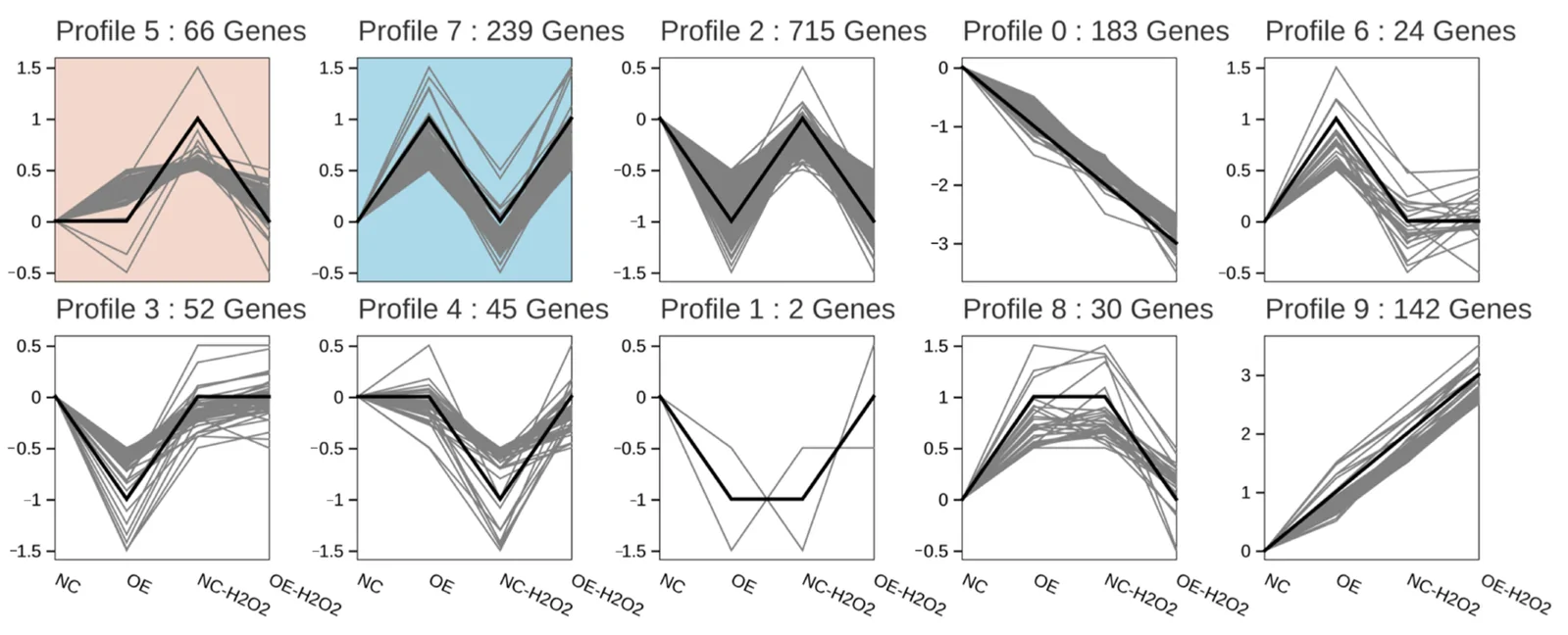
STEM trend clustering of differentially expressed genes across NC, OE-SOD1, NC + H_2_O_2_, and OE-SOD1 +H_2_O_2_ conditions. Each panel represents a distinct expression profile identified by STEM analysis. Gray lines indicate individual genes, and the black line represents the representative trend for each profile. Profile 7 reflects a basal SOD1 overexpression-associated transcriptional program, whereas Profile 5 represents an attenuated H_2_O_2_-inducible gene module in OE-SOD1 cells. The number of genes assigned to each profile is shown above each panel.

To resolve coordinated transcriptional programs across the four experimental conditions, including NC, OE-SOD1, NC + H_2_O_2_, and OE-SOD1 + H_2_O_2_, DEGs were subjected to STEM trend clustering. STEM analysis classified DEGs into multiple discrete expression profiles, representing recurrent expression patterns across the four conditions (Figure 5). Among the significant profiles, two major expression programs were particularly prominent.

Profile 7, comprising 239 genes, represented a basal OE-dominated program. Genes in this profile showed higher expression in OE-SOD1 cells than in NC cells under basal conditions, and this separation was maintained after H_2_O_2_ challenge. This pattern indicates a genotype-associated shift in the basal transcriptional state of DF-1 cells. Functional enrichment analysis of Profile 7 genes showed over-representation of cytokine/chemokine and innate immune-like signaling pathways, including cytokine–cytokine receptor interaction, JAK-STAT signaling, and NF-κB signaling. In parallel, Profile 5, comprising 66 genes, represented an attenuated H_2_O_2_-inducible program. Genes in this profile were strongly induced by H_2_O_2_ in NC cells but showed markedly weaker induction in OE-SOD1 cells under the same oxidative challenge, consistent with SOD1-dependent buffering of a subset of H_2_O_2_-responsive transcripts (Tables S5 and S6). Beyond these two modules, STEM clustering identified additional recurrent expression patterns, including profiles with monotonic decreases across the four conditions, such as Profile 0, monotonic increases, such as Profile 9, and transient responses associated with SOD1 overexpression or H_2_O_2_ exposure.

### 3.6. Functional Characterization of the Attenuated H_2_O_2_-Inducible Module in OE-SOD1 Cells

**Figure 6:**
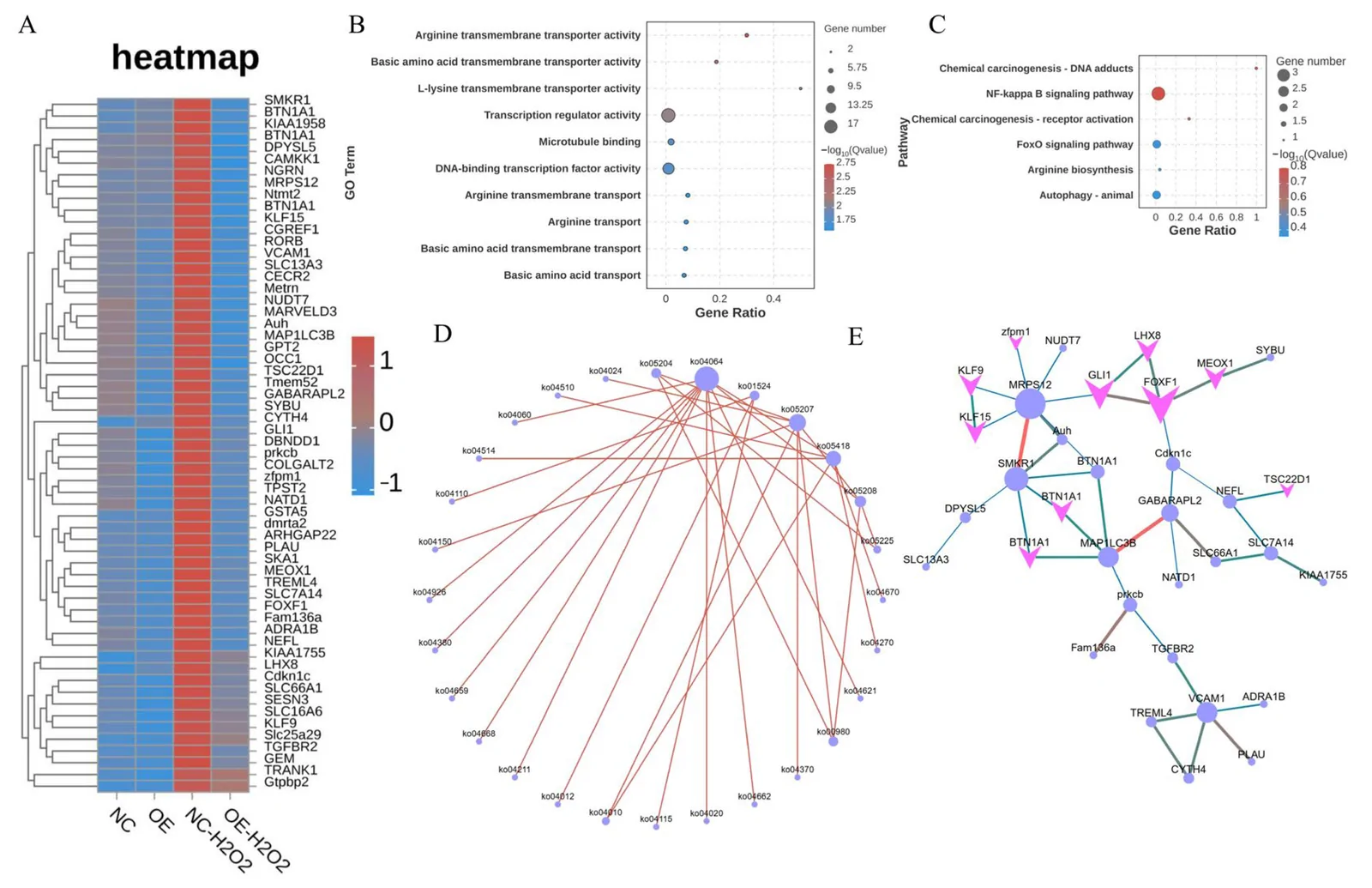
Profile 5 represents an attenuated H_2_O_2_-inducible transcriptional module in OE DF-1 cells. (**A**) Heatmap of Profile 5 genes across four experimental states (NC, OE, NC-H_2_O_2_, and OE-H_2_O_2_). Values are row-scaled (z-score) normalized expression, with hierarchical clustering of genes shown on the left. (**B**) GO enrichment of Profile 5 genes. Dot size indicates the number of genes mapped to each term, and dot color denotes −log10 (q value); the x-axis shows gene ratio. (**C**) KEGG pathway analysis of Profile 5 genes. (**D**) Network view of enriched KEGG pathways for Profile 5 genes, illustrating connections among terms based on shared mapped genes. (**E**) PPI network of Profile 5 genes (STRING-based). Node size reflects connectivity (degree), and edges represent predicted/known associations.

Among the significant STEM profiles, Profile 5, comprising 66 genes, displayed a typical attenuated H_2_O_2_-inducible trajectory across the four experimental conditions: NC, OE-SOD1, NC + H_2_O_2_, and OE-SOD1 + H_2_O_2_. Heatmap visualization showed that Profile 5 genes were strongly induced by H_2_O_2_ in NC cells, whereas their induction was markedly weaker in OE-SOD1 cells under the same oxidative challenge (Figure 6A).

GO enrichment analysis of Profile 5 revealed a focused functional signature dominated by basic amino acid transport. The enriched terms included arginine transmembrane transporter activity, basic amino acid transmembrane transporter activity, L-lysine transmembrane transporter activity, and related biological processes associated with amino acid transmembrane transport. In addition, Profile 5 was enriched for regulatory functions, including transcription regulator activity, DNA-binding transcription factor activity, and microtubule binding, indicating that this attenuated module contains both transport-associated and regulatory genes (Figure 6B).

KEGG analysis showed that Profile 5 genes were distributed across several stress- and metabolism-related pathways, including NF-κB and FoxO signaling, reactive oxygen species-associated pathways, autophagy, and arginine biosynthesis (Figure 6C). Consistently, the KEGG enrichment network showed interconnections among multiple enriched pathways, indicating coordinated pathway-level organization rather than isolated single-pathway effects (Figure 6D). Protein–protein interaction analysis further revealed a structured network within Profile 5, with MRPS12 and SMKR1 identified as major hub genes. A secondary submodule contained autophagy- and quality-control-related components centered on the ATG8-family proteins MAP1LC3B and GABARAPL2. Notably, SMKR1 was connected to MAP1LC3B through BTN1A1, suggesting potential cross-links between the hub-centered network and the autophagy-associated submodule (Figure 6E).

### 3.7. RT-qPCR Validation of Selected Genes

**Figure 7:**
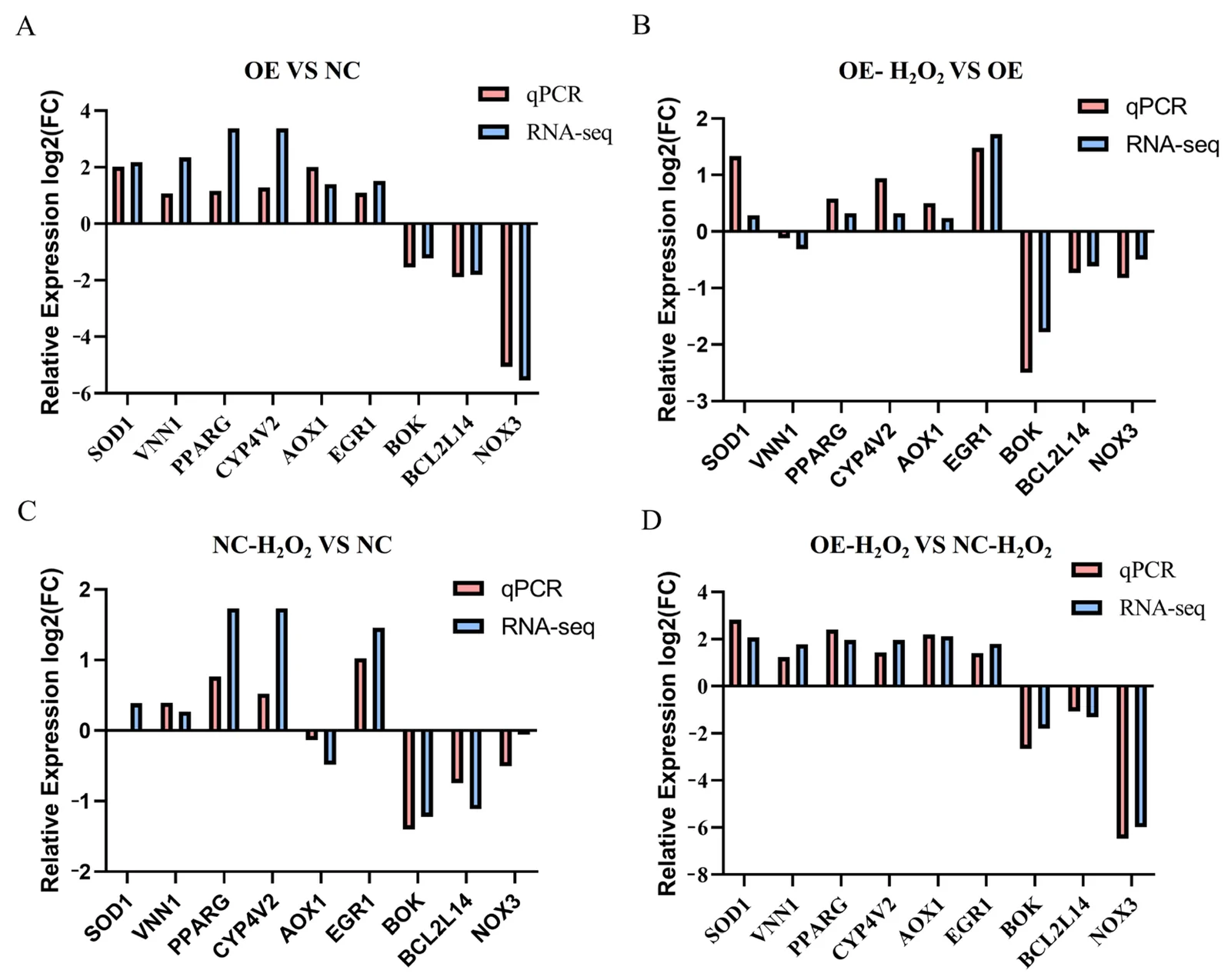
RT-qPCR validation of selected RNA-seq findings. RT-qPCR was performed to validate expression changes for nine representative genes (SOD1, VNN1, PPARG, CYP4V2, AOX1, EGR1, BOK, BCL2L14, and NOX3) and compared with RNA-seq-derived log2 fold changes. Panels show four contrasts: (**A**) OE vs. NC, (**B**) OE-H_2_O_2_ vs. OE, (**C**) NC-H_2_O_2_ vs. NC, and (**D**) OE-H_2_O_2_ vs. NC-H_2_O_2_. Bars represent log2 fold changes measured by RT-qPCR (pink) and RNA-seq (blue). RT-qPCR values were calculated using the 2^−ΔΔCt^ method with GAPDH as the reference gene.

RT-qPCR was performed to validate the RNA-seq results across four pairwise comparisons (Figure 7A–D). Overall, the RT-qPCR results showed expression trends consistent with those obtained from RNA-seq for all selected genes across the comparisons. In the baseline comparison between OE-SOD1 and NC cells, RT-qPCR confirmed increased expression of SOD1, VNN1, PPARG, CYP4V2, AOX1, and EGR1 in OE-SOD1 cells, whereas BOK, BCL2L14, and NOX3 were downregulated (Figure 7A). Under oxidative challenge, the OE-SOD1 + H_2_O_2_ versus OE-SOD1 comparison showed further induction of EGR1 and modest increases in PPARG, CYP4V2, and AOX1, while BOK, BCL2L14, and NOX3 remained downregulated; VNN1 showed only a minimal decrease (Figure 7B). In NC cells, the NC + H_2_O_2_ versus NC comparison revealed increased expression of PPARG, CYP4V2, and EGR1, accompanied by decreased expression of BOK, BCL2L14, and NOX3, while AOX1 showed a slight reduction (Figure 7C). Finally, in the OE-SOD1 + H_2_O_2_ versus NC + H_2_O_2_ comparison, RT-qPCR confirmed higher expression of SOD1, VNN1, PPARG, CYP4V2, AOX1, and EGR1 in OE-SOD1 + H_2_O_2_ cells, together with lower expression of BOK, BCL2L14, and particularly NOX3 (Figure 7D).

## 4. Discussion

In the present study, we investigated whether SOD1 overexpression enhances resistance to acute oxidative stress and alters the associated transcriptional response in chicken DF-1 cells. Our results showed that elevated SOD1 expression improved cellular viability during H_2_O_2_ challenge, markedly reduced necrotic cell death, and attenuated a subset of H_2_O_2_-inducible transcriptional programs. These findings support our central hypothesis that increasing antioxidant capacity through SOD1 not only limits oxidative injury but also modifies the broader cellular response to oxidative stress in an avian model. Together, the phenotypic and transcriptomic data provide complementary evidence that SOD1 contributes to oxidative-stress adaptation in chicken fibroblasts.

Oxidative stress is an important biological challenge in poultry production, where heat stress, pathogen exposure, nutritional imbalance, mycotoxins, and intensive production conditions can disturb redox homeostasis and impair cellular function [5,28,29]. Although the major components of antioxidant defense are evolutionarily conserved across vertebrates, birds and mammals differ in several physiological and metabolic characteristics relevant to redox regulation. Avian species generally exhibit high metabolic demands, yet many birds maintain relatively long lifespans and show distinctive patterns of oxidative damage and stress resistance compared with mammals [7,8]. Comparative studies using avian and mammalian fibroblasts have also reported differences in cellular resistance to oxidative and other stressors [9]. These observations suggest that findings obtained predominantly from mammalian systems cannot necessarily be assumed to fully represent oxidative-stress adaptation in avian cells. Therefore, direct characterization of antioxidant regulation in chicken cells is important for understanding redox biology in poultry.

Within the antioxidant defense system, the superoxide dismutase (SOD) family constitutes a major class of enzymatic antioxidants that catalyze the dismutation of superoxide radicals and thereby contribute to redox homeostasis. Among SOD isoforms, SOD1 has received particular attention due to its broad intracellular distribution and close association with cellular homeostasis [10]. Beyond its canonical role in ROS detoxification, SOD1 has also been implicated in oxidative-stress-related signaling pathways and transcriptional regulatory networks, suggesting that it may influence cellular adaptation to external stressors through both antioxidant and regulatory mechanisms [11,30,31]. In our study, stable SOD1 overexpression significantly increased DF-1 cell viability under normal culture conditions and markedly alleviated the H_2_O_2_-induced decline in viability (Figure 1E,F and Figure 2A–C). These results indicate that increased SOD1 abundance enhances the ability of chicken fibroblasts to tolerate acute oxidative challenge. This finding extends observations from mammalian models to an avian cellular system and supports an important role for SOD1 in the cellular antioxidant defense of chicken cells [12,32].

Oxidative stress can induce multiple forms of cell death, including apoptosis and necrosis, and the predominant death modality often depends on the severity and duration of oxidative insult as well as cell type [33,34,35,36]. In the present study, H_2_O_2_ treatment increased both apoptosis-associated nuclear changes and PI-positive membrane damage in DF-1 cells. However, the most prominent protective effect of SOD1 overexpression was a marked reduction in PI-positive necrotic cells, whereas the apoptosis-associated signal showed comparatively limited changes (Figure 2D–F). These findings suggest that SOD1 overexpression does not broadly suppress all forms of cell death, but preferentially reduces severe oxidative injury associated with loss of membrane integrity. Previous studies in DF-1 cells have also shown that H_2_O_2_ can induce substantial necrotic injury [33], supporting the biological relevance of this response in chicken fibroblasts.

The transcriptomic analysis further demonstrated that SOD1 overexpression reshaped the response of DF-1 cells to oxidative challenge. H_2_O_2_ treatment in NC cells induced extensive transcriptional remodeling and enriched several stress- and injury-related pathways, including p53 signaling, chemical carcinogenesis-reactive oxygen species, chemical carcinogenesis-DNA adducts, and MAPK signaling. These pathways are well-established components of the cellular response to oxidative damage [37,38,39]. Importantly, OE-SOD1 cells still exhibited clear transcriptional responses after H_2_O_2_ treatment, indicating that SOD1 overexpression did not eliminate cellular stress sensing. Instead, a subset of H_2_O_2_-responsive genes showed weaker induction in OE-SOD1 cells. This pattern suggests that elevated SOD1 expression buffers the magnitude of selected stress-responsive transcriptional programs rather than completely suppressing the oxidative-stress response.

STEM analysis provided further resolution of these transcriptional changes. Profile 5, comprising 66 genes, displayed a characteristic attenuated H_2_O_2_-inducible pattern, with strong induction in NC cells but markedly weaker induction in OE-SOD1 cells. Functional enrichment of this profile was dominated by arginine and basic amino acid transmembrane transport. Cationic amino acid transport is closely linked to nutrient availability, cellular stress adaptation, and survival [40,41], while arginine metabolism is connected to nitric oxide production, polyamine synthesis, immune regulation, and redox homeostasis [42]. The reduced induction of this module in OE-SOD1 cells may therefore reflect a lower requirement for stress-associated metabolic remodeling when antioxidant capacity is increased. However, because amino acid uptake and intracellular metabolite levels were not directly measured, these results should be interpreted as transcriptional associations rather than evidence that SOD1 directly regulates amino acid transport.

In addition, the PPI network of Profile 5 contained an autophagy-related submodule involving MAP1LC3B and GABARAPL2 (Figure 6E). Both proteins belong to the mammalian ATG8/LC3-GABARAP family and are closely associated with autophagosome formation, maturation, and autophagic flux [43]. LC3-family proteins, including LC3B, are commonly linked to autophagosome membrane elongation and autophagy monitoring, whereas GABARAP-family members, including GABARAPL2, have been implicated in later stages of autophagosome maturation and autophagosome–lysosome fusion [44]. Oxidative stress is known to interact closely with autophagy, which can function as a quality-control mechanism to remove damaged organelles and abnormal protein aggregates and thereby contribute to redox homeostasis. Therefore, the presence of MAP1LC3B and GABARAPL2 in the attenuated H_2_O_2_-inducible Profile 5 network suggests that SOD1 overexpression may influence oxidative stress-associated autophagy or protein quality-control responses [45]. However, because autophagic flux was not directly measured in this study, this interpretation should be considered hypothesis-generating and requires further validation using LC3-II accumulation, p62/SQSTM1 turnover, and autophagy flux assays.

RT-qPCR validation further supported the overall reliability of the RNA-seq results. SOD1 remained highly expressed in the OE background, confirming the stability of the overexpression model. NOX3 was markedly downregulated in SOD1-overexpressing cells. Because NADPH oxidases contribute to cellular ROS generation [46], this observation raises the possibility that increased SOD1 expression is associated not only with enhanced antioxidant capacity but also with changes in ROS-generating pathways. In parallel, altered expression of cell-death-related genes such as BOK [47] and BCL2L14 [48] suggests that SOD1 overexpression may also reshape the basal transcriptional state associated with sensitivity to oxidative injury. These expression patterns are consistent with the observed cytoprotective phenotype, although additional functional experiments will be required to establish direct causal relationships.

From an avian biology perspective, the present study extends the understanding of SOD1-mediated cytoprotection to a chicken cellular system and demonstrates that elevated SOD1 expression affects both cellular survival and stress-responsive transcription. This is relevant to poultry biology because oxidative stress is a common consequence of multiple environmental, nutritional, and disease-associated challenges encountered during production. Nutritional antioxidants, including selenium and vitamins E and C, as well as plant-derived antioxidant compounds, have been investigated as potential strategies to mitigate oxidative damage in poultry [4,49]. Our findings therefore provide a cellular and molecular framework that may support future studies aimed at understanding how antioxidant capacity influences stress resilience in avian species.

Several limitations should be considered when interpreting these findings. First, DF-1 cells are an immortalized chicken fibroblast cell line and therefore cannot fully reproduce the physiological complexity of oxidative-stress responses in primary tissues or whole animals. Second, H_2_O_2_ treatment represents an acute experimental oxidative challenge, whereas oxidative stress under poultry production conditions is often multifactorial and may occur chronically. Third, the present study did not directly compare chicken and mammalian cells under identical experimental conditions. Therefore, although our results establish SOD1-associated cytoprotection and transcriptional remodeling in an avian cellular context, they do not demonstrate that these mechanisms are unique to birds. Future studies using primary chicken cells, tissue-specific models, in vivo oxidative-stress conditions, and direct avian-mammalian comparisons will be important for determining which aspects of SOD1-mediated stress adaptation are broadly conserved and which may reflect avian-specific regulatory features.

## 5. Conclusions

This study demonstrates that SOD1 overexpression enhances resistance to acute oxidative stress and modifies stress-responsive transcription in chicken DF-1 cells. These findings support the role of SOD1 as an important component of antioxidant defense in an avian cellular context and extend current understanding of how increased antioxidant capacity can influence both cellular survival and transcriptional adaptation during oxidative challenge. The study therefore provides a molecular framework for further investigation of oxidative-stress regulation in poultry. Future studies using primary chicken cells, tissue-specific models, and in vivo oxidative-stress conditions will be important for validating the physiological relevance of these findings. Direct comparisons between avian and mammalian systems may also help distinguish broadly conserved antioxidant mechanisms from regulatory features that are more specific to avian species.

## Data Availability

The RNA-seq data reported in this study have been deposited in the China National Center for Bioinformation (https://www.cncb.ac.cn/; accessed on 27 August 2026), accession number: CRA044905.

## Supplementary Materials

The following supporting information can be downloaded at: https://www.mdpi.com/article/10.3390/antiox15091074/s1, Table S1: Primer sequences; Table S2: RNA-seq QC and mapping summary; Table S3: All DEGs; Table S4: STEM clustering output; Table S5: GO enrichment of profile 5 and profile 7; Table S6: KEGG enrichment of profile 5 and profile 7. Supplementary File S1. Uncropped Western blot images corresponding to Figure 1D.

## Abbreviations

The following abbreviations are used in this manuscript:

SOD1	Superoxide dismutase 1
ROS	Reactive oxygen species
DF-1	Chicken embryo fibroblast cell line
NC	Negative control
CCK-8	Cell Counting Kit-8
PI	Propidium iodide
RNA-seq	RNA sequencing
DEG	Differentially expressed gene
GO	Gene Ontology
KEGG	Kyoto Encyclopedia of Genes and Genomes
PCA	Principal component analysis
FPKM	Fragments per kilobase of transcript per million mapped reads
FDR	False discovery rate
PPI	Protein–protein interaction
STEM	Short Time-Series Expression Miner
RT-qPCR	Reverse transcription quantitative polymerase chain reaction
GAPDH	Glyceraldehyde 3-phosphate dehydrogenase
