# Supplementary material for: SOD1 Overexpression Alleviates H2O2-Induced Oxidative Stress and Reshapes Transcriptomic Responses in Chicken DF-1 Cells
Source: Antioxidants (Basel). 2026 Aug 27;15(9):1074. doi: 10.3390/antiox15091074 (PMC13603882; doi:10.3390/antiox15091074)
Supplement: Supplementary file 1 [file antioxidants-15-01074-s001.zip › Supplementary File S1. Uncropped western blot images corresponding to Figure 1D/Note.pdf]

1. GAPDH

Molecular weight: approximately 38 kDa

Lane order from left to right:

DF-1 control cells; DF-1 KO-SOD1 cells; molecular weight marker; DF-1 control cells; SOD1-overexpressing DF-1 cells.

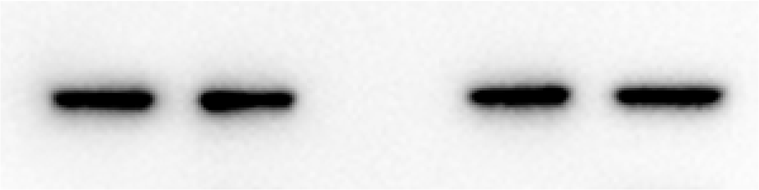

2. SOD1

Molecular weight: approximately 15 kDa

Lane order from left to right:

DF-1 control cells; DF-1 KO-SOD1 cells; molecular weight marker; DF-1 control cells; SOD1-overexpressing DF-1 cells.

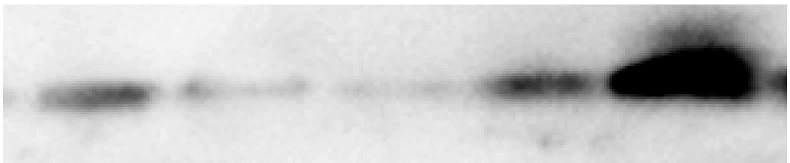

Note:

The **DF-1 KO-SOD1 sample shown in the original uncropped Western blot was not included in the experimental design or subsequent analyses of the present study**. This lane was present on the original membrane but was not used for the results reported in Figure 1D. Only the DF-1 control and SOD1-overexpressing DF-1 samples were used for the comparison presented in the manuscript.
